# Computed Tomography (Ct) Scan Assisted Machine Learning in the Management of Artifacts Related to Paranasal Sinuses and Anterior Cranial Fossa

**DOI:** 10.1155/2022/6993370

**Published:** 2022-10-07

**Authors:** Abdullah Musleh

**Affiliations:** King Khalid University, College of Medicine, Abha, Saudi Arabia

## Abstract

Computed tomography (CT), through the use of ionizing radiation, allows us to assess the different parts of the body. It is made up of an X-ray tube that rotates rapidly around the patient generating the radiation beam. This is attenuated with the patient producing information, which is collected by the detectors that are opposite to the tube located in the gantry (part of the tomography equipment); finally, these collected data are sent to the computer that will reconstruct the information obtained and will represent it as an image on the monitor. In the practice of a study, artifices or artifacts may appear regardless of their origin, which limits the scan examination; this leads to stopping the examination and starting again, and added to this with the contrast media, they have to apply these drugs again. State-of-the-art scanners allow complete reconstructions to be performed with few projections, limiting radiation doses, by means of statistical algebraic reconstruction methods. The present work shows the simulation of artifacts in sinusitis diagnosis computed tomography images, the extraction of features from each image, and an automatic classification algorithm for the differentiation of artifacts. The results show that the algorithm is able to classify the simulated artifacts with a percentage of 90%.

## 1. Introduction

**Figure 1:**
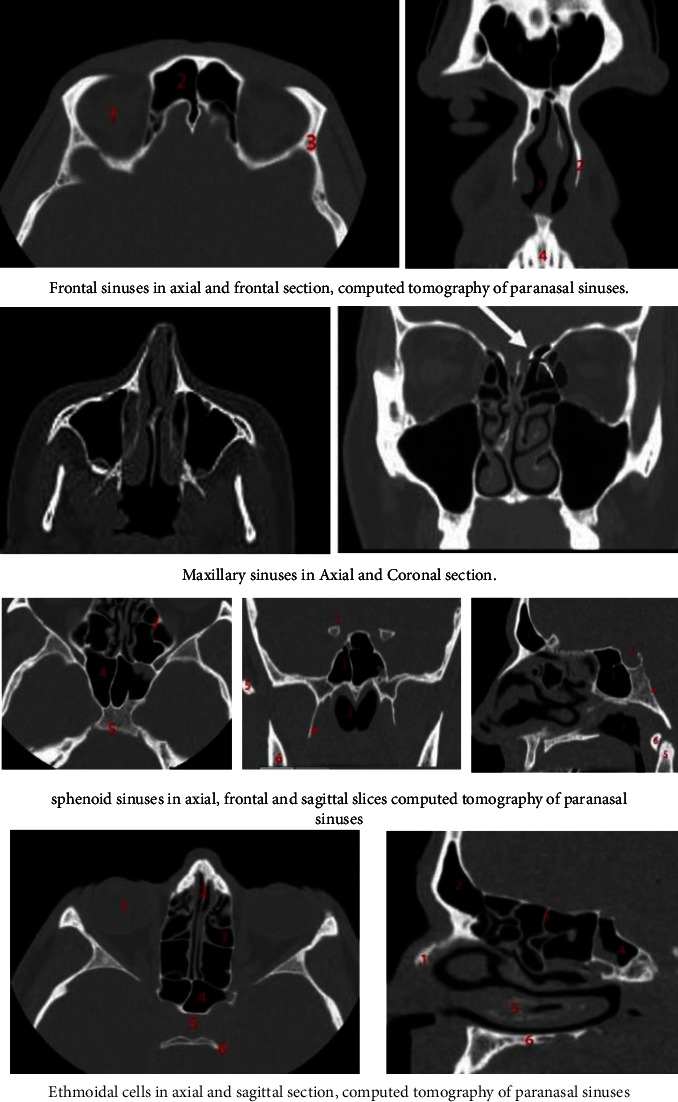
Various types of sinuses.

**Figure 2:**
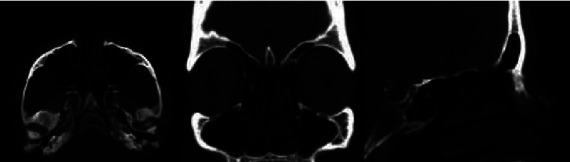
Tomography of the paranasal sinuses.

Computed tomography (CT), which employs ionizing radiation, enables us to evaluate various anatomical structures. The X-ray tube, which rapidly revolves around the patient and emits the radiation beam, is the main component [1]. This is diminished by the patient producing data, which is then gathered by detectors opposite the tube in the gantry (a component of the tomography equipment), and finally, these collected data are sent to the computer that will reconstruct the information obtained and will display it as an image on the monitor [2]. Sinusitis (Figure 1), a pathology marked by swelling and thickening of the mucosa lining the paranasal sinuses, may be brought on by obstructions, infections, or even anatomical variations [3]. Computed tomography is used frequently to diagnose sinusitis. It should be noted that sinusitis can affect both adults and children due to a viral or bacterial infection of the upper airways [4]. When we have an acute sinus infection, the tomographic study will reveal air-fluid levels because of the build-up of secretions that reduce the amount of air in the sinus and result in increased radiopacity and thickened mucosa. Chronic sinusitis is characterised by nonuniform, persistent radiopacity of the sinus, sclerosis, or thickening of the nearby bone, hypertrophy of the sinus mucosa, enlargement of the inferior turbinates, and nasal and sinus polyps [5]. The role of CT in this pathology makes it a tool that can be used directly in clinical practice because of the high sensitivity and specificity of contemporary technological equipment. Despite what has already been said, diagnosing the local sinusitis index is crucial. As a result, we are interested in figuring out how frequently patients have paranasal sinusitis (Figure 2), which can be diagnosed by CT scan [6].

*The signs of acute sinusitis*: Behind the skull, nose bridge and cheeks are air-filled cavities known as the sinuses. Ciliated cells that line the sinuses produce the mucus that covers them. Bacteria and other contaminants are kept from spreading by mucus and nose hairs. A mucus that has thickened due to sinusitis can prevent the sinuses from receiving mucus. Sinus pressure rises as fluid accumulates. From bacterial growth, walls become infected. This affects 14% of Americans. In many cases, triggers include nasal or throat viruses or bacteria [7].

A sinus infection's early stages can cause symptoms mistaken for a cold, making diagnosis challenging. Congestion and fatigue can be brought on by either. A cold goes away in 5 to 7 days, but sinusitis can persist for weeks if left untreated. Flu-like symptoms, a fever, and facial pain are all symptoms of sinus infections. A quick office exam, patient history, and physical examination all help to identify acute sinusitis. In addition to inspecting your ears, nose, and throat, the doctor may also palpate your face to feel for sinus tenderness while asking you about the severity and duration of your symptoms. In order to make a diagnosis, your doctor might put a nasopharyngoscope through your nose. Doctors may order X-rays and CT scans to examine the paranasal sinuses and rule out any syndromes or other conditions. In most cases, sinus infections are treated and resolved in less than three weeks. Long-term sinus infections last longer than three weeks [8].

The mucosa lining the paranasal sinuses is closely related to the mucous membranes that line the nostrils and is completely pneumatized. The nasal cavity is encircled by them, and they drain into it. They are situated around the facial and cranial bones. It serves to warm, humidify, and protect the air that is inhaled as well as the nearby bones from which it gets its name. It also protects the lungs in times of trauma [9]. They consist of the sphenoid sinus, ethmoidal cells, and pairs of frontal and maxillary sinuses. They originate from the diverticula in the nostril wall. The superior, middle, and inferior turbinates are bony shell-shaped structures located inside the nostrils on the side walls. They have a mucosal lining that helps to maintain the quality of the air we breathe. The nasal septum, which is made of bone and cartilage on the backside and separates the right and left nostrils, is a bony structure [10]. The ostium connects them to the nostrils, and their pseudostratified cylindrical and ciliated respiratory epithelium lining ensures their physiology. However, if there is any obstruction, the partial pressures of oxygen and carbon dioxide inside the lungs become out of balance (14).

In computed tomography, the process of reconstructing images involves effectively utilising the interaction of x-rays with the body, taking into account the projections obtained in each beam path and accounting for the data recorded by each detector [11]. The same plane is subjected to numerous projections in order to reconstruct the cross-section of the body part being studied. In addition to the incidence and interaction of the X-rays, which are deterministic by the beam of the ray and their respective projections according to the angle of rotation and their arrival at the detectors, the human body is composed of structures of various densities that are very different from one another, allowing the creation of a recognition pattern that aids in the processing of images. As computed tomography (CT) technology has advanced, significant aspects of image acquisition should be considered. One of the most significant is the decrease in scan time, which is necessary to improve image quality and reduce exposure doses while also shortening reconstruction times. Although the number of projections can be determined in TAC, it is still not necessary to implement a mathematical formula for its processing; instead, effective algorithms are applied to evaluate it. Johann Radon developed one of the first image reconstruction methods in 1917 [12], who used a mathematical solution through a transform, which indicates that an image is determined by an infinite set of its projections [13].

In the mathematical processes of image reconstruction, the statistical methods fulfil a complementary function to the analytical methods but are differential at the same time, being the iteration methods our object of study in the present work. To ensure the best possible development of image processing, computed tomography relies on both mathematical and analytical data processing techniques. However, these techniques must take into account factors that make tomography a superior method of diagnosis in medicine, such as image quality, acquisition and processing times, minimization of radiation dose, reduction of noise and artifacts, and computational costs. Image quality is the result of the application of all the processes listed above. Still, in clinical practice, the patient's condition may act as a source of artifice generation and involuntary movements [14].

These aspects change the quality of the image and its diagnosis, necessitating a repeat exam and subjecting the patient to an excessive dose of radiation. In addition, when contrast media are provided, the travel times in the images are slowed down, which can also affect the diagnosis. Through an iterative reconstruction by approximation, which allows collecting and retrieving images to complete the study, it is possible to finish the study without needing to repeat the examination. Despite this, these methods require a complex algorithm and the appropriate implementation of the data for its processing. When an image appears with artifacts from a previous image, it is automatically recognised and moves on to carry out the correction process without the need to restart the study.

## 2. Theoretical Framework

### 2.1. Machine Learning

Artificial intelligence is the branch of computing in which basic analog processes can be executed automatically in response to input with its respective output through programmed logic. Machine learning, as part of artificial intelligence, is responsible for generating algorithms that have the ability to learn, avoiding continuous programming; what is required is to feed the algorithm with data so that it learns and has autonomous decision alternatives. Machine learning is a scientific method that allows computers with the computational capacity to learn and extract patterns and correlate them by themselves. These patterns can then be used to predict behaviors that facilitate decision-making, and this is possible through information analysis called “training data” [15, 16].

### 2.2. Support Vector Machines (SVMs)

The decision surface is learned by an SVM using two input classes. The data are mapped to a higher-dimensional feature space using a Gaussian kernel or another kind of kernel. The SVM theory is based on structural risk minimization, and neural networks have been used to highlight applications of the theory that have demonstrated outstanding performance [17]. The SVM resolves a quadratic problem where the number of inputs or training data equals the number of coefficients. Many applications exist for classification and regression, including image classification, character recognition, pattern classification, function identification. Another definition of the SVM is a system for efficiently training linear learning machines[18].

#### 2.2.1. SVM for Regression

**Figure 3:**
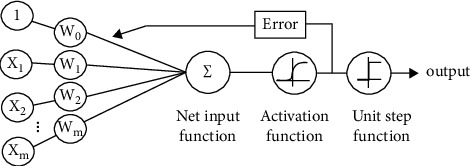
SVM architecture for regression taken from Acharya [20].

The goal of the SVM is to find a function *f*(*x*) that has at most one deviation *ϵ* from the output *y*_*i*_ for all the training data, and at the same time, that is as small as possible. What has not been considered are minor errors *ϵ*, only those greater [19]. What is sought with the SVM for regression (Figure3) is to perform a mapping of the training data *xϵX*, to a space of greater dimension *F* through a nonlinear mapping *φ*  : *XF* , where the linear regression can be performed. The support vectors on which all the data that contain the most information possible depend [17].

#### 2.2.2. SVM for Classification

**Figure 4:**
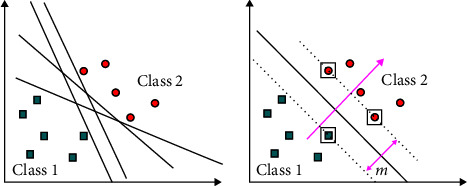
Linearly separable case [22].

To build a hyperplane that separates two classes, labelled *x*, 1, +1, so that the distance between the optimal hyperplane and the closest training pattern margin is the maximum [21]. Within the classification problems are the linearly separable and nonlinearly separable cases. Suppose we have *S* labeled points for training as shown in Figure 4.

### 2.3. Cross-Validation

Within the data validation techniques in machine training, it is obtained through theoretical physical models or with simulations through the hold-out validation methods and *k*-fold. When the amount of data for training and testing is limited, the hold-out method can be applied to estimate the error [23]; the objective is the adequate use of all the instances in *D* for the test training.

#### 2.3.1. Leave-One-Out Cross-Validation

**Figure 5:**
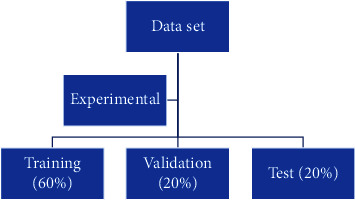
Hold-out separation.

It is based on a confusion matrix where one record is used to test the validation classifier *K* times to run the training algorithm through *N* iterations, excluding one from the sample process is repeated until leaving each of the samples outside the confusion matrix as shown in Figure 5.

#### 2.3.2. Cross-Validation *k* Iterations

To perform *k*-folds validation, we split the original dataset into *k* equal parts; then, during model training, we use the first *k* of these parts as the test set and the remaining (*k* − 1) parts as the training set. Each iteration of this procedure will collect data from a new test set [23].

### 2.4. ROC Curve

ROC curves are used to evaluate the performance of a classification method, which seeks to highlight event characteristics [24, 25]. The operating characteristics curve (receiver operating characteristics) represents the sensitivity and specificity for each threshold value and that allows comparing two or more classifiers based on their discriminant capacity, and we have the fraction of the true positive fraction (TPF) is plotted against the false positive fraction (FPF) which is given by the following function:

(1) $$\begin{array}{c} ROC_{\left(c\right)}=FTP_{\left(c\right)},TPF_{\left(c\right)};\;c\in -\infty ,\infty \text{.} \end{array}$$

One of the indices for estimating the efficacy of a system is the area under the ROC curve (AUC), whose value will always be between 0.5 and 1, which is why it is commonly used to compare the performance of the markers, the ROC provides a description of the separation between the distributions of positives and negatives.

## 3. Methodology

**Figure 6:**
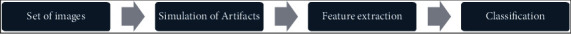
A proposed methodology for the simulation and classification of artifacts in CT images.

The methodology includes four steps in which a modification is made to a set of computed tomography images extracting features from said images using singular value decomposition, the training of a support vector machine with different kernels, and validation to find the validity of the proposed algorithm as shown in Figure 6.

### 3.1. Image Set

As input, a set of 43 paranasal sinus and skull base tomography images taken from different patients was used, where it was taken into account that the bone structures were differentiated. On the other hand, these images were found in the three body planes: sagittal, coronal, and axial.

### 3.2. Simulation of Artifacts

Taking into account that there is no tomography equipment to carry out tests on images with noise or real artifacts, it was decided to apply different algorithms that allow simulating these effects on tomography images. In practice, there are two types of artifices that can be presented in computed axial tomography; depending on the origin, we can classify them as direct or indirect:

- Direct: they are generated by the patient: voluntary and involuntary movements
- Metallic implants: osteosynthesis material, surgical clips
- Indirect: detector misalignment; beam hardening; undersampling

#### 3.2.1. Patient Movement

In Euclidean space, a rotation is represented by a special kind of matrix called a rotation matrix. Rotation matrices in two dimensions look like the following equation (equation (2)):

(2) $$\begin{array}{c} R\left(\theta \right)=\left(\begin{array}{cc} \cos\;\theta & -\sin\;\theta \\ \sin\;\theta & \cos\;\theta \end{array}\right)\text{,} \end{array}$$

where *θ* represents the angle of rotation of the matrix.

#### 3.2.2. Concentric Rings

Two or more circles are said to be concentric if they have the same centre but different radii. Assuming a point (*h*, *k*) as the centre and a radius of *r*, the equation of the circle is

(3) $$\begin{array}{c} {\left(x-h\right)}^{2}+{\left(y-k\right)}^{2}=r^{2}\text{.} \end{array}$$

Taking into account what is described in equation 31, rotation was used to generate different rings within the image with the same centre to simulate concentric rings' artifice.

#### 3.2.3. Beam Hardening

RGB values are converted to grayscale using the NTSC formula:

(4) $$\begin{array}{c} GrayImage=0.299R+0.587G+0.114B\text{.} \end{array}$$

The relative brightness of red, green, and blue light as perceived by the average person is well captured by this equation. The grayscale image was then converted to binary using Otsu's technique.

The threshold value is determined by Otsu's method in such a way that the spread within each segment is as small as possible while the spread between segments is as large as possible. To do this, the quotient between the two variances is calculated, and the maximum quotient is sought for a given threshold value. Let *p*(*g*) be the probability of occurrence of the gray value 0  <  *g*  <  *G* (*G* is the maximum gray value). Then, the probability of occurrence of the pixels in the two segments is

(5) $$\begin{array}{c} K_{0}:P_{0}\left(t\right)=\overset{t}{\underset{g=0}{\sum }}p\left(g\right),K_{1}:P_{1}\left(t\right)=\overset{t}{\underset{g=t+1}{\sum }}p\left(g\right)=1-P_{0}\left(t\right)\text{.} \end{array}$$

Once the image has been binarized, it is sought that the whites are attenuated to obscure the skull's internal structures. At this point, a threshold of 0.4 was used in order to detail such structures. Using the 43 original images, and taking into account that a transformation was applied to each of them in order to simulate the artifacts, a total of 172 images were obtained.

#### 3.2.4. Feature Extraction

Using the theory and considering the development, the null values of the matrix that are in the established range are discarded. When applying it to the 172 images, 172 matrices are obtained, and finally, the singular value of each element is obtained by passing each image through an algebraic method, which compresses the matrix that composes it. With the data obtained, a general matrix is fed that stores the result of the SVD of each image in order to train a machine learning algorithm in which interclass comparisons will be made with support vector machines and classification binary vectors.

### 3.3. Machine Learning

The implementation of the algorithm to develop programs or solve specific problems can result in recognizable or repetitive patterns within the image, with which patterns or some kind of task can be predicted, improving its performance. For this case, a machine learning approach will be applied that allows to select and determine, and within a computerized axial tomography study bank, the images have some artifice and manage to classify them autonomously with respect to the original image. Finally, a cross-validation method of *k*-folds cross-validation is used in order to train the machine and find the real values of the simulation of the artifacts with respect to the reference image.

## 4. Results

**Figure 7:**
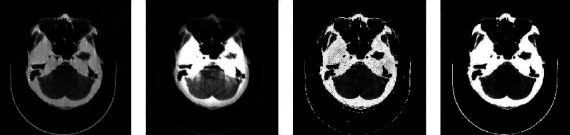
Orbit normal scan image, the same image with motion simulation.

Figure 7 shows the result of applying the rotation matrix to an image.

On the other hand, from left to right, a normal brain scan image is followed by the simulation of the incident ray beam hardening device, that is, when there is a loss of contrast with respect to the original image due to the excessive absorption of the beam of the ray by the bony structures of the skull. Finally, you can recreate double artifices and concentric rings on the right, which considerably limits their evaluation.

**Figure 8:**
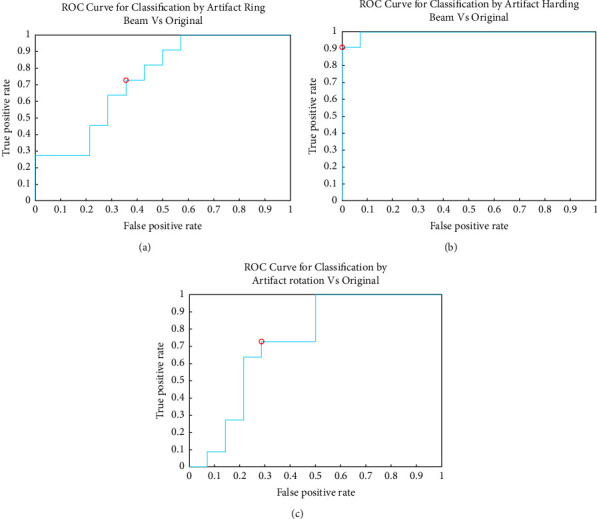
(a) ROC curve original image vs. concentric rings; (b) ROC curve original image vs. artifact hardening beam; (c) ROC curve original image vs. artifact rotation.

In Figure 8(a), by means of the ROC curve, it can be seen that the AUC value (area under curve) is approximately 0.78, which indicates that this artifact has an error range of 12% with respect to the original images. This value was obtained through 30 iterations of the model, which can vary by 0.06 between iterations of the same cut.

It is important to mention that the samples or class must be of the same matrix size, which is ensured by the singular value decomposition (SVD); even so, the images correspond to different sections and brain structures, so the Kernel is of the Gaussian type.

In the case of cross-validation *k*-folds, between the original images and the images with beam hardening artifacts, the value is approximately 0.91 AUC, again demonstrating that it corresponds to a true positive; we can say that images are supported as a method of validation and classification. These were also performed in 30 iterations with a variation between each exercise and 0.09 between samples. The result can be seen in Figure 8(b). In the last case study, an AUC value of 0.63 (Figure 8(c)) was obtained, which is somewhat low compared to previous studies, but it is still considered a true positive, and this is due to although the rotated image maintains the same matrix size, the values of each composition pixel vary depending on the degree of rotation of the image. The result varies between 0.09 between the samples after 30 iterations.

With the values from the different samples, validations, and binary classifications between training samples and classes, it is possible to programme an algorithm that automatically identifies artifice. From this, machine learning's importance in image reconstruction as a method of learning and predicting them, optimising tomography imaging, can be seen (CT). As seen in Figures 8(a)–8(c), the trend of the tests is in the upper left corner, indicating that it has great prediction accuracy with each sample. We can also see that the model can distinguish between normal and fake images.

The device with the best test performance was the incident ray beam hardening with 83% efficiency, followed by the concentric ring device with 79% and the rotation device with 73%. This is because the distortion of the original image varies depending on the artifice, with beam hardening causing the least distortion and rotation causing the most. Each artifice's simulation is based on a package of images. Due to the number of iterations and because they cannot be classified linearly, polynomial or Gaussian Kernel types are used when performing different tests. Even so, training the machine learning algorithm for image classification with CT artifacts is reliable. The case study will use a confusion matrix to redistribute the 172 images into 4 groups of 43 each. With these values, we can calculate the model's classification accuracy through the following formula:

(6) $$\begin{array}{c} Precision=\frac{TP}{\left(TP+FP\right)}\text{,} \end{array}$$

where TP is True Positive, and FP is False Positive.

This means that 80% of the images will be recognised within or derived from the original image.

In the second validation measure, the Recall (Completeness) will be calculated, which refers to the prediction capacity of the machine learning model.

(7) $$\begin{array}{c} Recall=\frac{FN}{\left(TP+FN\right)}\text{,} \end{array}$$

where TP is True Positive, and FN is False Negative.

This means that the model is able to recognise 90% of the images with artifice and the normal images.

Finally, the *F*-score or *F*1 will be calculated to determine the positive predictive value, and the performance of the model is the following equation:

(8) $$\begin{array}{c} F-Score=\frac{Precision\times Recall}{\left(Pres+Recall\right)\times 2}\text{.} \end{array}$$

**Figure 9:**
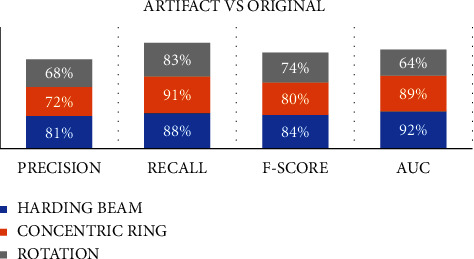
Final results of the classification algorithm with an interclass comparison between the simulated artifacts and the original image.

This indicates that the quality of the model is approximately 84% reliable, taking into account the entire sample, that is, the 172 images. This can be seen in the graphs of each ROC curve of the artifacts versus the original images, where each score is close to 1 as a true positive. What proceeds next is to carry out the same exercise, but with each of the proposed tricks against the set of original images to obtain the following Figure 9.

The data are taken in each of the cases, and it is evident that the percentages are relatively consistent in comparison with those obtained through the classification model of prediction proposed in MATLAB; on the other hand, for each artifact, it can be concluded that the artifact that most closely resembles the original image is hardening of the incident beam since the original image is maintained, but the intensity of the grayscale is affected, showing overexposure in the brain structures is consequently poorly defined. The second artifice with greater adherence to the original image is that of concentric rings, which preserves the original format but the distortion due to overlapping is evident in the brain structures. Lastly, the images with a rotation device are the ones that lose the most similarity with the original images, where the evaluation of any brain structure is totally limited.

## 5. Discussion of Results

The image quality is improved by the algebraic methods' significant reduction of all types of noise and artifacts. Another benefit of this method is that reconstruction can be completed without requiring all of the conventional projections of a typical study, which also results in a significant decrease in exposure doses per study. For example, when using iterative algorithms, matrices are recalculated in all possible directions to obtain new sinograms. When using other statistical methods, corrections are made in the geometry using the incident ray beam approach, which also requires more computational work and, as a result, has a higher GPU cost [20–22]. From the foregoing, we can infer the followings:

- High contrast and superior quality images
- Requires fewer projection data for image reconstruction
- Considerable reduction in exposure dose
- Longer reconstruction time
- High computational costs

Hybrid architectures that use both reconstruction methods and parallel algorithms are being developed to create more efficient reconstruction mechanisms. Higher image resolution means a larger initial matrix and more equations per pixel, which equals more tomograph detectors. Parallel algorithms and filtering techniques such as Gaussian, median, Wiener, and bilateral filters are being implemented to improve data acquisition efficiency [21].

As we have discussed, several ways to “clean” an image efficiently with statistical methods require complex adaptation to current hardware systems. It works with GPU systems, a graphic processing unit, to speed up iterative data processing, implement new algorithms, and achieve maximum reduction [17, 20, 21]. Statistical methods have the advantage of not requiring a large number of projections and data to reconstruct later. Noise and artifacts in images are not only caused by external factors, such as voltage arcs, improper instrument calibration, and misaligned detectors. Image quality improves with analytical and mathematical algorithms.

## 6. Conclusions

Any artifice chosen for the study can be duplicated from any scanned image, replicating a real medical image while testing the visualisation of sinusitis's important aspects. MATLAB could mimic the most common CT artifacts without changing the original matrix's dimensions. Based on the algebraic properties of each matrix, which can be transformed and used in other matrix operations, it was possible to duplicate tomography artifacts. Medical image classification algorithms are not linear due to matrix features when using normal and verification photos as training references. Artificially produced images require a Gaussian or polynomial Kernel to solve and represent. These photos can't be arranged on a normal plane for classification; hence, a linear solution isn't possible. The Gaussian Kernel allows features derived from artifact-filled simulated images to be consistent with the binary classification space defined; i.e., inside the classification group, we have two alternative labels between 0 and 1 to define positive or negative.

Using this binary classification, we can show how true positives and false positives behave in the sample. Machine learning helps computed axial tomography equipment's image reconstruction processes automatically recognise artifices and perform the appropriate modifications to lessen or eliminate image distortion. *K*-folds fit SVM classification data better, allowing model validation with an AUC (ROC). Cross-validation (*K*-folds) assesses the estimator's performance to determine the algorithm's prediction effectiveness. Iterations are used to retrain the model. Beam hardening artifact drastically altered the image, allowing for sensitive classification. This shows that the binarization algorithm's threshold affects image disturbance.

## Data Availability

The data used to support the findings of this study are included within the article.
